# Defining the Locus of Dopaminergic Dysfunction in Schizophrenia: A Meta-analysis and Test of the Mesolimbic Hypothesis

**DOI:** 10.1093/schbul/sbx180

**Published:** 2017-12-28

**Authors:** Robert McCutcheon, Katherine Beck, Sameer Jauhar, Oliver D Howes

**Affiliations:** 1 Department of Psychosis Studies, Institute of Psychiatry, Psychology & Neuroscience, King’s College London, London, UK; 2 MRC London Institute of Medical Sciences, Hammersmith Hospital, London, UK; 3 Institute of Clinical Sciences, Faculty of Medicine, Imperial College London, London, UK; 4 South London and Maudsley NHS Foundation Trust, London, UK

**Keywords:** PET, neuroimaging, nigrostriatal, F-DOPA, amphetamine

## Abstract

**Background:**

Studies using positron emission tomography to image striatal dopamine function, have demonstrated that individuals with schizophrenia display increases in presynaptic function. Mesolimbic dysfunction specifically, has previously been suggested to underlie psychotic symptoms. This has not been directly tested in vivo, and the precise anatomical locus of dopamine dysfunction within the striatum remains unclear. The current article investigates the magnitude of dopaminergic abnormalities in individuals with schizophrenia, and determines how the magnitude of abnormality varies across functional subdivisions of the striatum.

**Methods:**

EMBASE, PsychINFO, and MEDLINE were searched from January 1, 1960, to December 1, 2016. Inclusion criteria were molecular imaging studies that had measured presynaptic striatal dopamine functioning. Effects sizes for whole striatum and functional subdivisions were calculated separately. The magnitude of difference between functional subdivisions in patients and controls was meta-analyzed.

**Results:**

Twenty-one eligible studies were identified, including 269 patients and 313 controls. Individuals with schizophrenia (Hedges’ *g* = 0.68, *P* < .001) demonstrated elevated presynaptic dopamine functioning compared to controls. Seven studies examined functional subdivisions. These demonstrated significant increases in patients compared to controls in associative (*g* = 0.73, *P* = .002) and sensorimotor (*g* = 0.54, *P* = .005) regions, but not limbic (*g* = 0.29, *P* = .09). The magnitude of the difference between associative and limbic subdivisions was significantly greater in patients compared to controls (*g* = 0.39, *P* = .003).

**Conclusion:**

In individuals with schizophrenia dopaminergic dysfunction is greater in dorsal compared to limbic subdivisions of the striatum. This is inconsistent with the mesolimbic hypothesis and identifies the dorsal striatum as a target for novel treatment development.

## Introduction

Dysfunction of the dopamine system is one of the most well established findings in schizophrenia.^1–4^ Initial evidence was mostly indirect: based on preclinical work, the behavioral effects of drugs, and post-mortem studies.^5^ The development of positron emission tomography (PET) and single-photon emission computed tomography (SPECT), allowed the dopamine system to be studied in vivo in individuals with schizophrenia.^6^ Initial studies employed ligands specific to dopamine receptors, and allowed the quantification of receptor availability, while later work was able to investigate dopamine synthesis and release, and other aspects of dopaminergic function. Previous meta-analyses of these imaging studies have found that the major dopaminergic abnormality in schizophrenia is increased presynaptic activity in the striatum.^1,3^ While an elevation of postsynaptic D2 receptors has also been proposed, meta-analytic findings have been less convincing,^1^ although the presynaptic results raise the possibility that receptor differences may be masked by increased endogenous dopamine levels.^7–9^

**Fig. 1.**
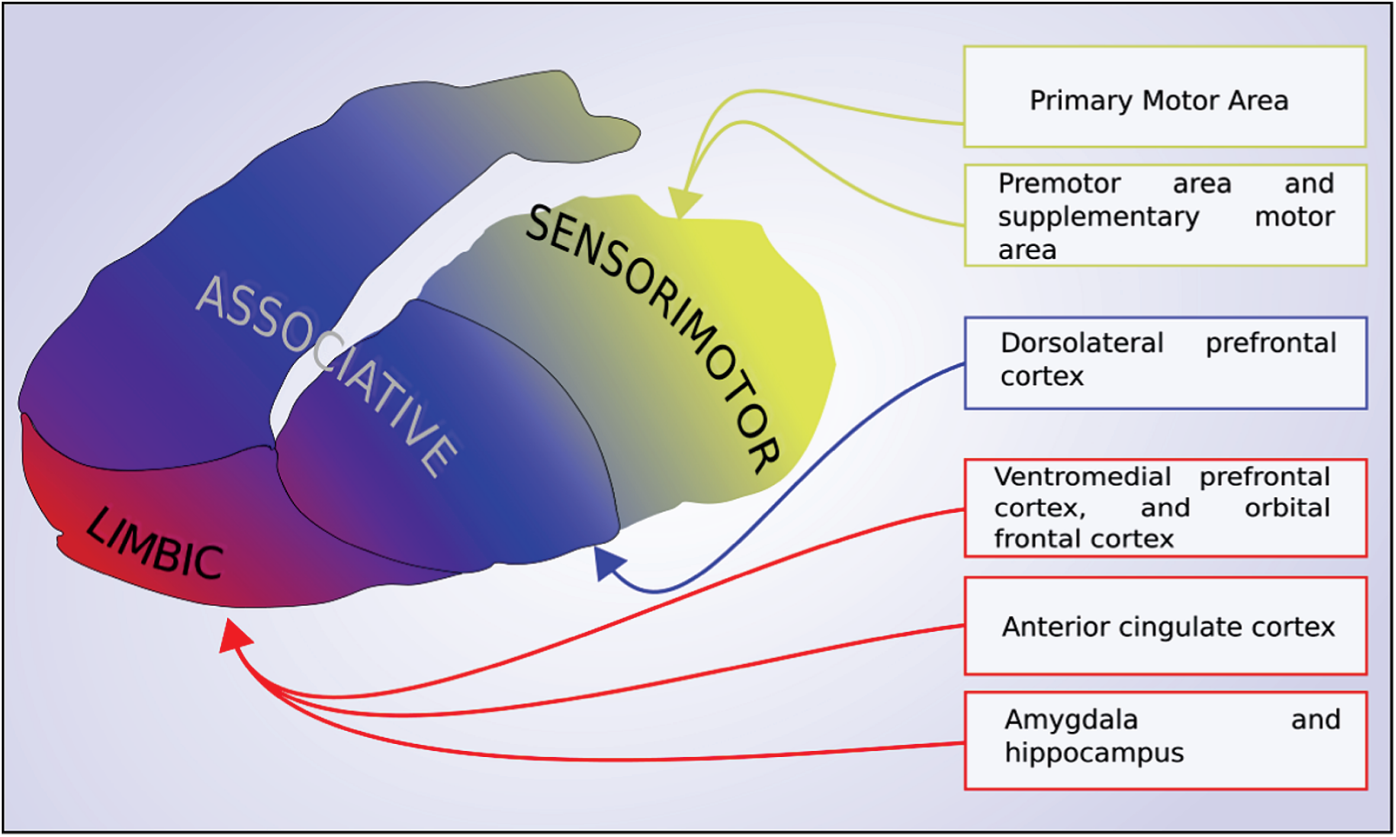
The topography of cortical afferents to the striatum illustrating the functional subdivisions.

Although cortical dopaminergic functioning has also been studied in schizophrenia,^10,11^ the main anatomical focus for investigations of dopamine dysfunction has been the striatum. Animal research has demonstrated that the striatum can be divided into 3 distinct subregions based on function and the predominant topography of brain projections from limbic, associative, and sensorimotor cortical areas to the striatum (figure 1).^12,13^ The antero-ventral striatum receives projections from limbic areas such as the orbital frontal cortex and medial temporal lobe, and consequently has been termed the limbic striatum. Anatomically it comprises the nucleus accumbens, and ventral parts of the caudate and putamen. The associative striatum, involved in higher cognitive function, receives projections primarily from cortical regions involved in executive and other higher cognitive processes, such as the dorsolateral prefrontal cortex, and is made up of the majority of the caudate, and the precommisural putamen. Finally, the sensorimotor striatum, involved in sensory and motor processing, receives afferent projections predominantly from sensory, motor, and premotor areas and consists of the postcommisural putamen. More recent imaging studies have indicated that this topography is paralleled in the human brain.^14,15^

Primarily based on preclinical research, dopaminergic hyperactivity of the limbic striatum has long been hypothesized as underlying psychotic symptoms.^16–19^ In vivo evidence for a specific mesolimbic abnormality has, however, been lacking. Initial imaging studies did not have sufficient resolution to visualize these subdivisions, and so reported values for either the whole striatum, or the anatomical divisions of caudate and putamen. However, improvements in PET cameras over the past decade have subsequently allowed dopaminergic function to be measured in these functional subdivisions. Work undertaken by Laruelle, Mawlawi, Martinez and colleagues,^20,21^ defined these subregions based on anatomical landmarks to allow the consistent reporting of subdivision findings in PET studies, and Howes, Egerton and colleagues determined the reliability of this approach.^22^ Initial studies using these functional divisions suggested that the greatest abnormality was within the associative striatum.^8,23^ Several further studies have since been performed, but the results have not been meta-analyzed.

In the current article, we aim to test the mesolimbic hypothesis by comparing the magnitude of dysfunction between the limbic and other striatal subdivisions. We also provide an update to previous meta-analyses of striatal dopamine function given that a significant number of studies have been published since previous reviews.

## Methods

EMBASE, PsychINFO and MEDLINE were searched from 1960 (or 1974 in the case of EMBASE), to December 31, 2016. Titles and abstracts were searched for the words: (“schizophrenia” or “psychosis” or “schizophreniform”) AND (“Positron Emission Tomography” or “PET” or “Single photon emission tomography” or SPET or “Single Photon Emission Computed Tomography” or SPECT) AND (Dopamine).

For the meta-analysis of presynaptic dopamine function in schizophrenia the inclusion criteria were: (1) studies of patients with schizophrenia diagnosed in accordance with criteria specified in the Diagnostic and Statistical Manual for Mental Disorders (DSM), or the International Classification of Diseases (ICD)^24,25^ and a control group; (2) reporting molecular imaging measures of presynaptic dopaminergic function (see supplementary methods for further details) for both the patient and control groups; (3) providing data enabling the estimation of mean difference between control and clinical groups for the dopaminergic measure; and (4) For the subdivision analysis only studies reporting all 3 subdivisions (limbic, associative, and sensorimotor subdivisions) were included to enable comparisons across regions.

Studies reporting data on dopaminergic functioning in individuals with treatment resistant schizophrenia, or co-morbid substance dependence, were excluded. This is because the primary neurobiological abnormality in these patients may not involve striatal hyperdopaminergia.^26–29^

### Data Extraction

The primary outcome of interest was the dopamine imaging parameter reported for the patient and control groups. For studies using labeled L-DOPA this was the influx constant in the region of interest relative to uptake in the reference region, while for studies using a release or depletion paradigm this was percent change in binding potential. In addition, author, year of study, number of participants, participant age and gender, illness duration, antipsychotic treatment, symptom scores, scan length, and whether an arterial input function was used were extracted.

Two studies^23,30^ reporting data in individuals with schizophrenia were not included due to sample overlap with Howes et al 2013.^31^ Where values for the whole striatum were not given but data for the caudate and putamen were reported, whole striatum values were calculated as described previously^1^ by weighting these values by their volumes as reported in the Oxford-GSK-Imanova Structural–Anatomical Striatal Atlas (43% and 57% respectively). If the ventral striatum was also reported the following weightings were used to derive a summary outcome for the whole striatum: caudate – 36%, putamen-putamen – 48%, ventral striatum – 16%.^32^

### Statistical Analysis

All statistical analyses were carried out using the “metafor” package (version 1.9–9) in the statistical programming language R (version 3.3.1). A minimum of 3 studies was required for meta-analysis. Standard effect sizes (Hedges’ *g*) for individual studies were estimated. The individual study effect sizes were then entered into a random effects meta-analytic model using restricted maximum likelihood estimation. *I*^2^ values were calculated to estimate between study heterogeneity. Where there were at least 10 studies included in a meta-analysis, funnel plots were constructed and visually inspected, and Egger’s regression test performed to check for the possibility of publication bias.^33^ Secondary subgroup and meta-regression analyses were undertaken to investigate the relationship between dopaminergic function and antipsychotic treatment (studies where ≥75% of patients were antipsychotic naïve were grouped as studies of predominantly antipsychotic naïve patients), scan length, paradigm type, modeling techniques, patient age and severity of symptoms.^34^ The statistical significance of differences between subgroups was tested for by fitting separate random effects models for each subgroup, and then comparing the subgroup estimates in a fixed effects model with a Wald-type test. A significance level of *P* < .05 (2-tailed) was used for all analyses.

To test the hypothesis that dopamine dysfunction is primarily located in limbic regions we first determined if there was a significant difference between patients and controls for each individual subdivision. We next calculated the magnitude of subdivision differences within group, and then determined whether the size of these differences significantly differed between groups (see below and supplementary information for further details).

In order to contrast and quantify the degree of dysfunction between subdivisions, a meta-analysis of difference was undertaken. In this we performed an inter-group (patient vs control) comparison of the magnitude of intra-group subdivision differences (eg, associative vs limbic). This approach employs methods used to quantify the propagation of errors.^35^ For each study, mean within subject differences in presynaptic function between subdivisions were calculated for both patient and control groups. For example, for patients the mean difference between associative and limbic measurements $\left({\bar{P}}_{\text{al}}\right)$ equals:

$$\begin{array}{c} {\bar{P}}_{\text{al}}={\bar{P}}_{a}-{\bar{P}}_{l}\\ \left({\bar{P}}_{a}=\text{mean associative value}\right)\\ \left({\bar{P}}_{l}=\text{mean limbic value}\right) \end{array}$$

In order to calculate the standard deviation of this mean difference, a correlation coefficient for presynaptic functioning between subdivisions is required (see supplementary information and supplementary eFigure 1 for full methods).^36^ We estimated this correlation coefficient from individual data for 37 subjects (21 controls and 16 individuals with schizophrenia).^37^ This showed Pearson’s coefficients of 0.72, 0.84, and 0.87 for correlations between sensorimotor-limbic, associative-limbic, and associative-sensorimotor divisions respectively. To be conservative the lowest of these values (0.72) was used for all comparisons. For example, to calculate the standard deviation of the limbic-associative difference in a patient group^35^:

$$\begin{array}{c} \sigma_{P}{}_{{}_{al}}=\sqrt{\sigma_{P_{a}}^{2}+\sigma_{P_{l}}^{2}-2r_{al}\sigma_{P}{}_{{}_{a}}\sigma_{P}{}_{{}_{l}}}\\ \sigma_{P}{}_{{}_{al}}=\text{Standard deviation of limbic}-\text{associative difference}\\ \sigma_{P_{a}}=\text{Standard deviation of associative subdivision values}\\ \sigma_{P_{l}}=\text{Standard deviation of limbic subdivision values}\\ r_{\text{al}}=\text{Correlation between limbic and associative subdivision values} \end{array}$$

We repeated the exercise to calculate the control mean difference $\left({\bar{C}}_{\text{al}}\right)$, and standard deviation $\left(\sigma_{C_{\text{al}}}\right)$, and then calculated the combined standard deviation of both groups $\left(\sigma_{{\text{PC}}_{\text{al}}}\right)$.

$$\sigma_{PC_{\text{al}}}=\sqrt{\frac{(n_{P}-1)\sigma_{P_{\text{al}}}^{2}+(n_{C}-1)\sigma_{C_{\text{al}}}^{2}}{n_{P}+n_{C}-2}}$$

*n*_*P*_ = Number of patients

*n*_*C*_ = Number of controls

The between groups effect size for the study was then calculated for each subdivision using this standard deviation as follows:

$$\text{ES}=\frac{{\bar{P}}_{\text{al}}-{\bar{C}}_{\text{al}}}{\sigma_{PC_{\text{al}}}}$$

This was converted to the bias corrected Hedges *g*,^38^ which was then entered into the standard meta-analytic model described above. For further information regarding methods see supplementary information.

## Results

A total of 1798 papers were identified. 21 of these met inclusion criteria (PRISMA flow diagram in supplementary eFigure 2).

### Studies of the Whole Striatum

**Fig. 2.**
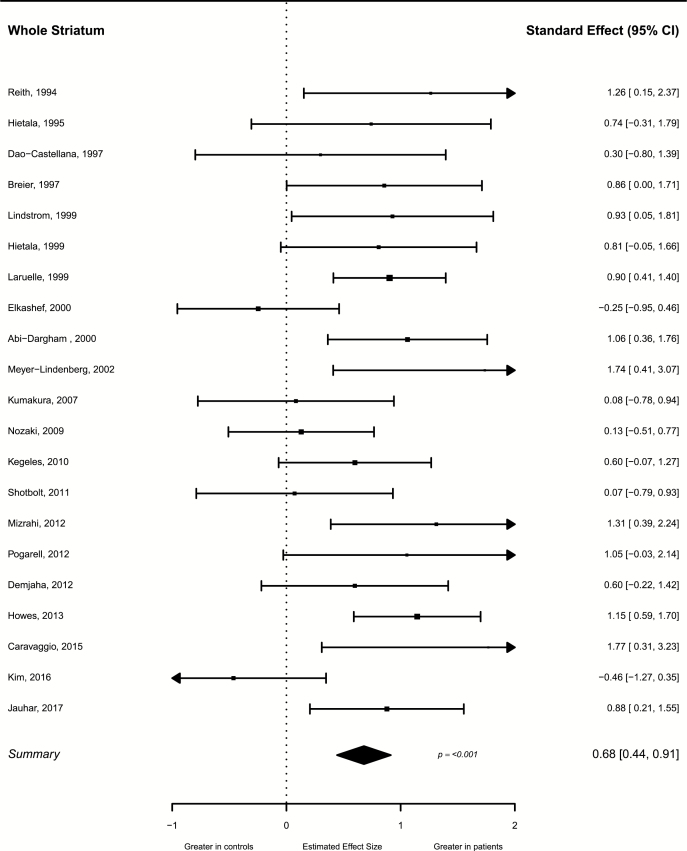
Forest plot of studies investigating presynaptic dopaminergic function in the whole striatum for individuals with schizophrenia. The forest plot shows the effect size (hedges *g*) and 95% CI for the difference between patients and controls. There is a significant elevation in schizophrenia with a summary effect size of 0.68.

21 studies of individuals with schizophrenia met inclusion criteria (see table 1 for study details). The studies included a total of 269 patients (256 with a diagnosis of schizophrenia, 3 schizoaffective disorder, and 10 a mixture of schizophrenia/ schizophreniform disorder) and 313 controls. Presynaptic dopamine function was significantly elevated in individuals with schizophrenia relative to controls with a summary effect size of 0.68 (see figure 2, 95% CI 0.44–0.91; *P* < .001). Egger’s regression test was not significant (*z* = 1.21, *P* = .23), indicating publication bias was unlikely. Visual inspection of the funnel plot potentially suggested asymmetry (supplementary eFigure 3), but a trim and fill analysis did not indicate any missing studies. The *I*^2^ value was 42.5%, suggesting a low to moderate level of heterogeneity. Subgroup meta-analysis of studies of predominantly drug naïve patients, and of patients who were receiving antipsychotic treatment, found a greater effect size in drug naïve patients (*g* = 0.78, *P* < .001 and .64, *P* < .001 respectively, see supplementary eFigure 5) but this difference was not statistically significant (*P* = .59). Studies using a challenge or depletion paradigm (*g* = 0.95, *P* < .001) showed a greater effect size when compared to those using labeled L-DOPA (*g* = 0.52, *P* < .001), and this difference was statistically significant (*P* = .049, see supplementary eFigure6). Neither scan time (*P* = .44) nor the use of an arterial input function (*P* = .55) was significantly associated with magnitude of effect size in the labeled L-DOPA studies. Meta-regressions of effect sizes against age (*P* = .29), total symptoms (*P* = .16), and positive symptoms (*P* = .39) were not significant.

**Table 1.** Studies of Presynaptic Dopamine Function in Individuals With Schizophrenia

	Controls		Patients								Scan Details	
Study	*N*	Age Mean (SD)/yr	*N*	Age	Diagnosis	Illness Duration/ mo	Antipsychotic Treatment	Total Symptom Score	Positive Symptom Score	Negative Symptom Score	Outcome Measure	PET Tracer and Method
Reith 1994^39^	13	36(13)	5	38(4)	Scz	168	4 naïve, 1 free >3 yr	PANSS 58	PANSS 14(3)	PANSS 12(2)	K_3_	[^18^F]DOPA
Hietala 1995^40^	8	27 (7)	7	26 (7)	Scz	24	All naive	PANSS 81(14)	na	na	K_i_	[^18^F]DOPA
Dao-Castellana 1997^41^	7	25 (5)	6	26 (9)	Scz	72	2 naïve 4 free ≥4 mo	PANSS 94 (na)	PANSS 21 (12)	PANSS 33 (7)	K_i_	[^18^F]DOPA
Breier 1997^42^	12	29.2 (9.0)	11	32.4 (10.0)	Scz	79.2	4 naïve, 7 free for >14 d	BPRS 28.8 (7.2)	BPRS 6.7 (2.8)	na	% Δ BP _ND_	[^11^C]Raclopride AMPH challenge
Hietala 1999^43^	13	30.4 (9.4)	10	29.6 (8.8)	7 Scz 3 SczAf	7	All naïve	PANSS 77.6 (na)	Na	na	K_i_	[^18^F]DOPA
Lindström 1999^44^	10	n/a	12	31(na)	Scz	31	12 naïve, 2 drug free > 2 yr	na	na	na	K_i_	[^11^C]DOPA
Laruelle^a^ 1999^45^	36	40 (9)	34	40 (9)	Scz	na	7 naïve, 27 free mean 104 d	na	17.5 (6.2)	16.8(6.6)	% Δ BP _ND_	[^123^I]IBZM AMPH challenge
Elkashef 2000^46^	13	34.6 (10.8)	19	36.3 (na)	Scz	207.6	10 medicated 10 drug free	na	na	na	uptake ratio:str/ref	[^18^F]DOPA
Abi-Dargham 2000^9^	18	31 (8)	18	31 (8)	Scz	na	8 naïve, 10 free for mean 139 d	66.6	18.2 (6)	13.8(5.4)	% Δ BP _ND_	[^123^I]IBZM AMPT
Meyer Lindenberg 2002^47^	6	34 (na)	6	35 (na)	Scz	na	All free ≥ 6 wk	na	na	Na	K_i_	[^18^F]DOPA
Kumakura 2007^48^	15	37.3 (6.4)	8	37.3 (6.3)	Scz	na	3 naïve, 6 free for ≥ 6 mo	PANSS 80.2 (4.7)	PANSS 15.4 (3.5)	PANSS 23.6 (4.0)	$K_{\text{in}}^{\text{app}}$	[^18^F]DOPA
Nozaki 2009^49^	20	35.1 (9.5)	18	35.6 (7.4)	Scz	26.4	14 naïve, 4 free	PANSS 79.2 (21.4)	PANSS 22.6(7.3)	PANSS 17.1(6.5)	K_i_	[^11^C]DOPA
Kegeles 2010^8^	18	29 (7)	18	29 (8)	Scz	na	6 naïve, 4 free ≥1 yr, 8 free for ≥20 d	PANSS 78.6 (20.6)	PANSS 21.7 (7.1)	PANSS 17.1 (5.9)	% Δ BP _ND_	[^11^C]Raclopride AMPT depletion
Shotbolt 2011^50^	20	43 (12)	7	43 (12)	Scz	na	All medicated	PANSS 56.8 (25.4)	PANSS 13.5 (6.7)	PANSS 15 (4.9)	K_i_	[^18^F]DOPA
Pogarell 2012^51^	7	23.6 (2.7)	8	25.4 (5.8)	Scz	24	Free for 1 wk	PANSS 76(18)	na	na	% Δ BP _ND_	[^123^I]IBZM AMPH challenge
Mizrahi 2012^52^	12	26.1 (3.83)	10	24.1 (5.0)	Scz/ Sczform	na	All naïve	na	PANSS 19.0 (3.8)	na	% Δ BP _ND_	[^11^C]-(+)-PHNO MIST
Demjaha 2012^26^	12	44.0 (11.9)	12	44.2 (8.9)	Scz	194.4	All medicated	PANSS 50.7 (5.8)	PANSS 11.9 (2.4)	na	K_i_	[^18^F]DOPA
Howes^b^ 2013^31^	29	29.3 (7.5)	29	33.7 (10.6)	Scz	na	16 medicated, 8 free, 5 naïve	CASH 77.6 (47.6)	CASH 38.3 (30)	CASH 31.9 (22.9)	K_i_	[^18^F]DOPA
Caravaggio 2015^53^	10	29.1 (8.4)	3	30 (16)	Scz	na	All medicated	na	na	Na	% Δ BP _ND_	[^11^C]-(+)-PHNO AMPT depletion
Kim 2016^54^	12	30.3 (8.4)	12	31.1 (9.8)	Scz	111.3	All medicated	PANSS 50.3 (11.1)	PANSS 10.8 (2.7)	PANSS 13.2 (5.2)	K_i_	[^18^F]DOPA
Jauhar 2017^37^	22	24.5 (4.5)	16	26.3 (4.4)	Scz	24	11 naïve, 3 free	PANSS 72.9 (16.5)	PANSS 17.8(6.3)	PANSS 18.8(4.1)	Ki	[^18^F]DOPA

*Note*: AMPH, amphetamine; AMPT, alpha-methyl-para-tyrosine; BP, Binding Potential; BPRS, Brief Psychiatric Rating Scale; CASH, Comprehensive Assessment of Symptoms and History; Ki, utilization rate constant of DOPA relative to a reference region; ${\text{K}}_{\text{in}}^{\text{app}}$, net blood-brain DOPA clearance; MIST, Montreal Imaging Stress Test; na, not available; PANSS, Positive and Negative Syndrome Scale; Ref, reference region; Scz, schizophrenia; Sczform, Schizophreniform; SczAf, Schizoaffective disorder; Str, striatum.

^a^Includes all subjects from Laruelle et al^55^ and Abi-Dargham et al.^56^

^b^Includes the entire sample from McGowan et al.^30^

### Studies of Limbic, Associative, and Sensorimotor Subdivisions

Seven studies of individuals with schizophrenia reported associative, sensorimotor and limbic subdivisions. These reported data on a total of 104 patients with schizophrenia (schizophrenia or schizophreniform disorder), and 174 controls. All 7 studies used the subdivision definitions proposed by Mawlawi et al,^21^ and Martinez et al.^20^

**Fig. 3.**
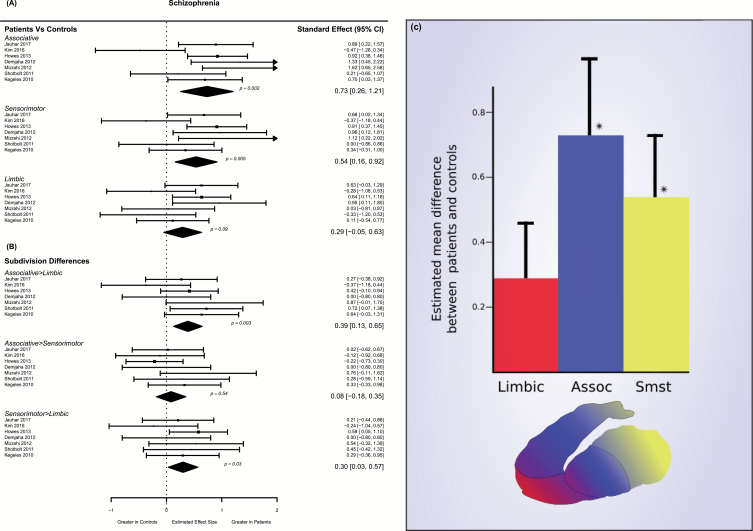
Studies of presynaptic dopamine function in individuals with schizophrenia by functional subdivisions. Significant elevations are seen for the associative and sensorimotor, but not limbic subdivisions. In schizophrenia the associative-limbic and sensorimotor-limbic differences are significantly greater in patients than in controls. (A) Effect size and 95% CI of difference in dopamine function in schizophrenia between patients and controls showing significant elevations in patients in associative (*g* = 0.73) and sensorimotor (*g* = 0.54) subdivisions but not limbic. (B) Effect sizes and 95% CIs of subdivision differences in schizophrenia between patients and controls. Patients show significantly greater associative-limbic (*d* = 0.38) and sensorimotor-limbic (*d* = 0.29) differences compared to controls. (C) Magnitude of patient-control differences in presynaptic dopamine functioning for striatal subdivisions in individuals with schizophrenia (*P < .05 for patient-control comparison), error bars represent 1 SE).

Significant differences were found between patients and controls for associative (schizophrenia – *g* = 0.73, *P* = .002) and sensorimotor (schizophrenia – *g* = 0.54, *P* = .009) subdivisions, but not for the limbic subdivision (schizophrenia – *g* = 0.29, *P* = .09) (see figures 3A–C). The results for the associative subdivision showed the greatest heterogeneity (*I*^2^ = 58.3%), with sensorimotor (*I*^2^ = 37.7%), and limbic subdivisions (*I*^2^ = 29.5%) showing relatively low levels of heterogeneity.

In individuals with schizophrenia, the difference between associative and limbic subdivisions was significantly greater in patients compared to controls (see figure 3C; effect size, *g* = 0.38, *P* = .004). Presynaptic dopaminergic function in schizophrenia was also significantly greater in the sensorimotor compared to the limbic subdivision compared to the difference in controls (*g* = 0.29, *P* = .03). There were no significant patient-control differences as regards the comparisons between dopamine function in the associative and sensorimotor subdivision (*g* = 0.08, *P* = .55). These comparisons showed low levels of heterogeneity (associative-limbic *I*^2^ = 25.5%, other comparisons *I*^2^ = 0).

## Discussion

Our main finding is that individuals with schizophrenia display greater elevation in dopaminergic functioning in the dorsal (sensorimotor and associative) relative to limbic striatum compared controls (figure 3). Moreover, there was no significant difference in presynaptic dopaminergic functioning between patients and controls for the limbic subdivision. This is, to our knowledge, the first study to meta-analyze differences between functional subdivisions of the striatum. Our analysis of the whole striatum included 8 additional studies published since previous reviews but is consistent with their findings in showing an increase in schizophrenia.^1,3^

### Methodological Considerations

Moderate heterogeneity was seen in the studies of individuals with schizophrenia. Methodological factors such as differences in the resolution of scanners, measurement time, experimental paradigm, and modeling technique may contribute to this heterogeneity. In addition, differences in the clinical characteristics of patients could contribute to between study heterogeneity, given findings that increased dopaminergic activity is linked to acute psychosis.^45,57,58^ Some studies included antipsychotic treated patients. However, our sub-analysis in antipsychotic free/naïve patients showed no statistically significant difference between these groups, and the elevation in presynaptic dopamine function was numerically larger in naïve patients than in antipsychotic treated patients, indicating antipsychotic treatment is unlikely to account for the elevation we see. Moreover chronic antipsychotic treatment may reduce dopamine synthesis capacity in some patients.^59^

We combined studies using challenge and depletion paradigms with those using radiolabeled DOPA. While there is some evidence that results from challenge paradigms are directly related to results from radiolabeled DOPA studies,^60,61^ it should be recognized that these measures are indexing different, albeit related, aspects of dopaminergic function, and could be influenced by different factors. Interestingly our sensitivity analysis found that effects were greater for the challenge/depletion studies (supplementary efigure 6), which could suggest that these aspects of the dopamine system are particularly affected in schizophrenia.

Another factor contributing to heterogeneity could be the inclusion of individuals with treatment resistant schizophrenia, or with co-morbid substance dependence, given recent findings these groups may show *reduced* presynaptic dopamine functioning.^26,28,29,62^ While we excluded studies specifically including these patients, many studies pre-dated these recent findings and did not specify these as exclusion criteria. As such it is likely that some of the included studies may have contained treatment resistant patients; indeed 2 studies report including patients taking clozapine.^9,46^ However this would, if anything reduce effect sizes given treatment resistant patients do not seem to show presynaptic dopamine elevation.^26,54^

We examined the *difference* between subdivisions, as, in the absence of individual patient data, this measure can be more accurately estimated than the *ratio* between subdivisions. A potential drawback of our measure is that if, eg, associative values are greater than limbic values, then a uniform proportionate increase in dopaminergic function across the whole striatum in the clinical group would lead to a greater absolute increase in the associative striatum, and thus give a larger associative-limbic difference. In our case, however, only 2 of the 7 control groups had a value for the associative region that was greater than the limbic value.^31,37^ Therefore, if anything, effects related to general increases in striatal functioning would reduce the magnitude of our findings.

When examining the differences between subdivisions, the assumed correlation between subdivisions has an influence on the precision of the estimated magnitude of difference between subdivisions, with a stronger correlation leading to larger effect sizes. The correlation coefficient we employed, however, was conservative, using the lowest of the correlation coefficients between subdivisions that we found in individual participant data. Using the largest coefficient of 0.87 gave an effect size of 0.50 (*P* = .01 for associative limbic measure, and 0.29 (*P* = .01) for the sensorimotor-limbic measure (supplementary efigure 5). Thus, the differences we report may underestimate the magnitude of the true difference.

The limbic striatum has a smaller volume than either the associative or sensorimotor subdivisions. As a result it is more susceptible to partial volume effects whereby its true activity may be diluted by spill over and spill in from adjacent regions.^63^ However, given that there is no consistent evidence of reduced limbic striatal volumes in schizophrenia this would be expected to affect measures in patients and controls equally.^64–67^ Moreover one study employed partial volume correction and found a significant elevation in the associative striatum, but not in the limbic striatum in schizophrenia and clinical high risk groups relative to controls,^52^ consistent with our meta-analytic findings. The fact that measures of dopamine functioning in the limbic striatum may be less reliable compared to measures in other subdivisions does mean, however, that it is possible the reduced limbic effect size (figure 3A) could be at least partially due to the increased noise inherent in measuring this region.^22,68^ This possibility is supported by some^26,52^ (but not all^31,37,54^) studies where the variance of the limbic measure, is noticeably greater than the variance of the associative measure.

Neither partial volume effects, nor reduced signal-to-noise, however, would account for the patient-control differences found when examining subdivision differences directly (figure 3B). In this case we are, eg, looking at limbic-associative differences in patients, and comparing this to the limbic-associative differences in controls. A reduction in signal-to-noise for the limbic measure will therefore affect patient and control findings equally, and will not bias the results. This means that while the reduced reliability of limbic measurements may increase the risk of a false negative, in this specific analysis it will not increase the likelihood of a false positive.

### The Anatomical Locus of Dopaminergic Dysfunction in Psychosis

Our meta-analysis confirms, using a larger sample, the previous meta-analytic findings of increased presynaptic dopamine functioning in schizophrenia in the striatum.^1^ Moreover, our meta-analysis extends understanding of the nature of dopamine dysfunction in psychosis by showing that the degree of dopaminergic dysfunction varies across the striatum, and identifies the dorsal striatum as the predominant locus of dopamine dysfunction in psychosis. Although patients showed no significant alteration in the limbic striatum relative to controls, we cannot rule out the possibility of a small difference in this subdivision. Nevertheless, in patients the dorsal to ventral balance was significantly shifted dorsally in patients when compared to controls. While a small mesolimbic abnormality may exist, overall these findings are not consistent with a hypothesis which proposes that the predominant locus of dopamine dysfunction is the limbic striatum.

Our findings thus suggest that models highlighting a primary role for excessive mesolimbic dopamine transmission in psychosis may need to be revised.^12–14,29^ The associative subdivision receives dopaminergic innervation from the substantia nigra,^12^ suggesting that nigrostriatal pathways may be disrupted in schizophrenia. This hypothesis is in keeping with findings of increases in some,^31,48^ although not all,^10^ aspects of dopamine functioning within the substantia nigra in schizophrenia. The elevation was greatest in the associative striatum, although this was not significantly greater than the elevation in the sensorimotor striatum.

It should be noted, that while our findings support the hypothesis that dopaminergic functioning within the associative striatum may be abnormal in schizophrenia, this does not preclude the possibility that the primary site of dysfunction exists in another brain region.^5^ The associative part of the dorsal striatum receives projections predominantly from dorso-lateral prefrontal cortex.^13^ Thus the dorsal locus of dopamine abnormality is consistent with the hypothesis that frontal cortical dysfunction underlies striatal dopamine abnormalities,^16,69^ although causality remains to be established in clinical studies.

Our findings also question the proposal that mesolimbic selectivity is a desirable property for pharmacological treatments of schizophrenia,^70^ and suggest instead that selectivity for the dorsal, particularly associative, striatum may show advantages in both efficacy and tolerability. Treatment strategies may be able to make use of the neurochemical distinctions found across striatal subdivisions. For example, dopamine transporter densities are greater in the ventral, compared to dorsal, striatum.^71^ Due to this variable distribution, combination therapy with a dopamine reuptake inhibitor and D2 antagonist could potentially reduce dopaminergic neurotransmission to a greater degree in the dorsal, as opposed to ventral striatum. There are potential risks to this approach, but evidence suggests that in some patients it may have benefits for the amelioration of negative symptoms.^72^

In conclusion, current molecular neuroimaging studies suggest that in individuals with schizophrenia the major locus of dopamine dysfunction is the dorsal striatum, and significant elevations were not seen in the limbic striatum. These findings are inconsistent with the mesolimbic hypothesis of schizophrenia, and suggest treatments showing nigro-striatal rather mesolimbic selectivity may have better efficacy and tolerability.

## Supplementary Material

Supplementary material is available at *Schizophrenia Bulletin* online.

Supplementary Data

## Funding

This work was supported by Medical Research Council-UK (no. MC-A656-5QD30), Maudsley Charity (no. 666), Brain and Behavior Research Foundation, and Wellcome Trust (no. 094849/Z/10/Z) grants to O.D.H., a Wellcome trust (no. 200102/Z/15/Z) grant to R.M., and the National Institute for Health Research (NIHR) Biomedical Research Centre at South London and Maudsley NHS Foundation Trust and King’s College London. The views expressed are those of the author(s) and not necessarily those of the NHS, the NIHR or the Department of Health.
