# Supplementary material for: Defining the Locus of Dopaminergic Dysfunction in Schizophrenia: A Meta-analysis and Test of the Mesolimbic Hypothesis
Source: Schizophr Bull. 2017 Dec 28;44(6):1301–11. doi: 10.1093/schbul/sbx180 (PMC5933516; doi:10.1093/schbul/sbx180)
Supplement: Supplementary Data [file sbx180_suppl_supplementary_data.doc]

**SUPPLEMENTARY DATA**

*The measurement of presynaptic dopamine functioning*

Methods for measurement of presynaptic dopamine function included the use of either radiolabelled L-dopa, or a dopamine receptor ligand in combination with a release or depletion paradigm. Radiolabelled L-dopa provides a measure of presynaptic dopamine synthesis capacity.^1,2^ Amphetamine stimulates dopamine release from neuron terminals, and inhibits its reuptake to increase extracellular dopamine levels.^3^ This can be quantified by measuring the degree to which amphetamine induced dopamine release displaces postsynaptic D2/3 receptor radioligands.^4,5^ A similar method can be employed to measure the magnitude of dopamine release in response to a psychological stress.^6^ Conversely, the administration of the dopamine depleting agent alpha methyl-paratyrosine, allows intrasynaptic levels of dopamine to be deduced, by measuring the increase in D2/3 radioligand binding following dopamine depletion.^7,8^ Together, these paradigms can be taken as measures of presynaptic dopamine functioning.^9^

*Calculating whole striatal values from caudate and putamen values*

A number of papers did not report whole striatal values but only anatomical subdivisions of caudate, putamen and ventral striatum. In these cases a whole striatal value was calculated based on previously reported methods.^9^ Volume based weightings were derived from the Oxford-GSK-Imanova Structural–anatomical Striatal Atlas to give weights of 0.43 and 0.57 respectively.^10^ If ventral striatum was also reported we used weights of 0.48, 0.36 and 0.16 for the caudate, putamen and ventral striatum respectively. A correlation of 0.7 for
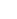
 was used (as in the analysis of difference of functional subdivisions). If it was necessary to combine left and right caudate/putamen, when the bilateral result was not reported, a correlation coefficient of 0.85 was used. Whole striatum standard deviation was calculated as follows:


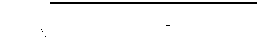


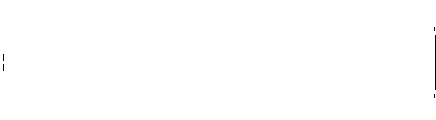


*Calculating Effect Sizes for Subdivision Differences*

In order to quantify the difference in dopaminergic alterations between subdivisions a meta-analysis of difference was undertaken. This involved calculating for each study mean within-group differences for subdivisions, and then contrasting patient and control groups against one another. eFigure 1 illustrates the measurements used. The mathematical basis of this comparison is well established and is based upon the concept of propogation of variance.^11^ Below follows an illustrative example, comparing associative and limbic subdivisions:

**1.** We first quantified the within group difference in subdivision means for an individual study:

For patients the mean difference between associative and limbic measurements (
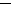
:


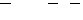


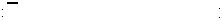


(
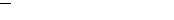


**2.** The standard deviation of this difference (
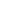
) can be calculated as follows:


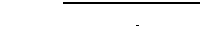


This requires the calculation of the correlation coefficient ‘
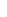
’ between presynaptic functioning in the various subdivisions.^12^ Examination of individual patient data from Jauhar et al^13^ (in press) showed Pearson’s coefficients of 0.72, 0.84, and 0.87 for correlations between sensorimotor-limbic, associative-limbic, and associative-sensorimotor respectively. The lowest (i.e. most conservative) of these values (0.72) was used for all comparisons.

Repeating the exercise for the controls allows the calculation of the control mean difference (
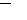
, and standard deviation (
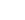
) .

**3.** The following steps are simply those used to calculate abetween groups effect size in the usual manner. In the current study this was performed in R using the *metafor* package. The values calculated above allow for the calculation of the combined standard deviation of both groups:


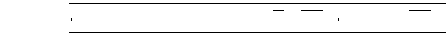


Which allows for the calculation of the between groups effect size for the study:


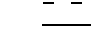


This can be bias corrected in the usual manner to provide Hedges g, which can be then entered into the standard meta-analytic model used previously.


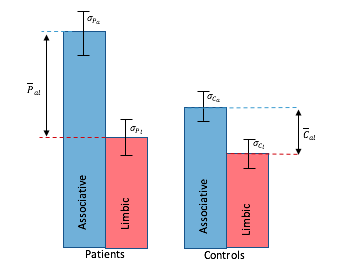


**eFigure 1:** Values used in the meta-analysis of difference. For illustrative purposes only – in reality the limbic values are typically larger than the associaitive values for controls.

Additional records identified

(n=7)

Records identified in database search

(n=1798)

Records screened

(n=1805)

Duplicates

(n=799)

Conference Abstracts

(n=375)

Not suitable

(n=610)

Eligible studies included in review

(n=21)

**eFigure 2.** Flow diagram illustrating study selection

**
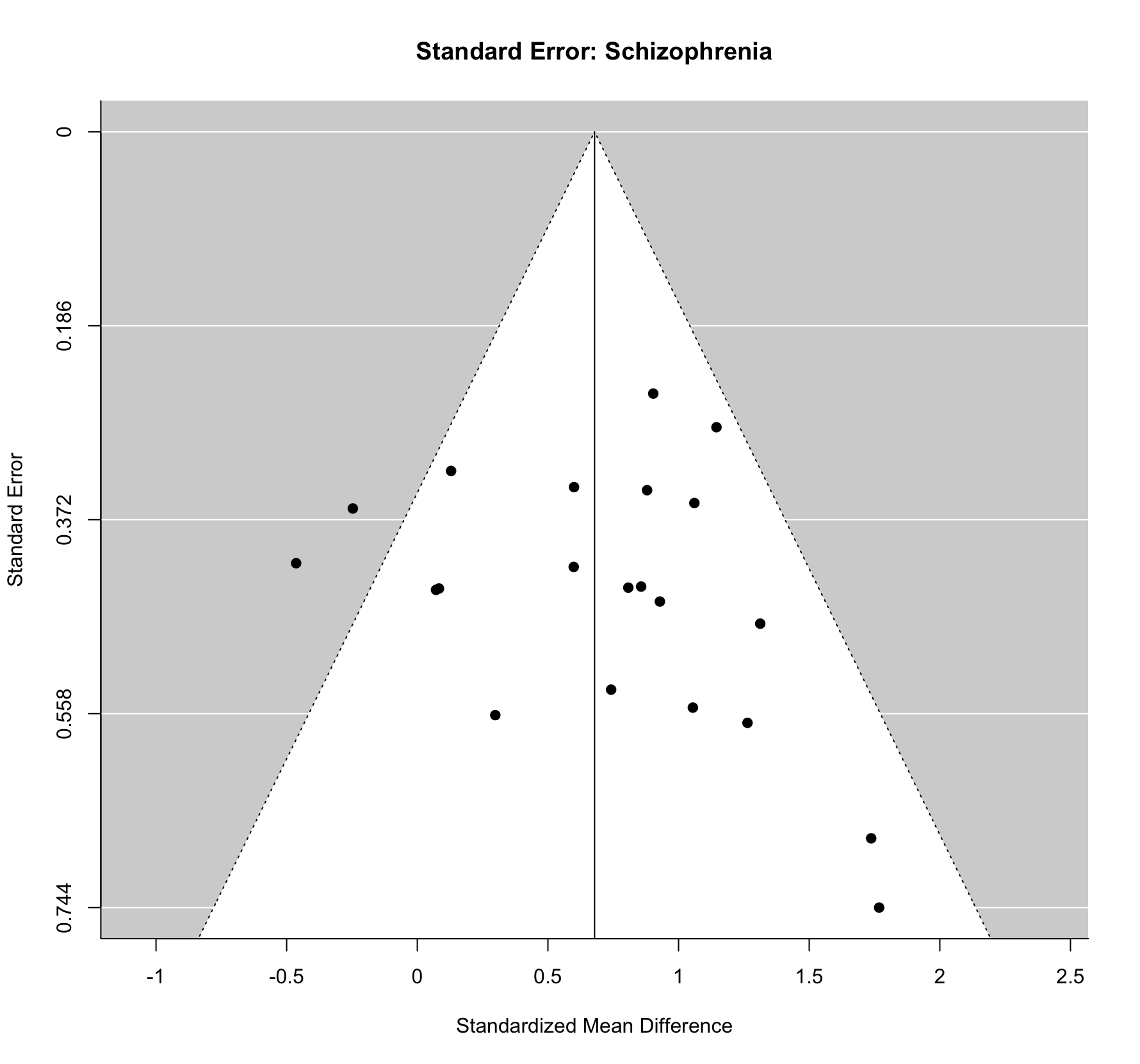
**

**eFigure 3.** Funnel plot for studies of presynaptic dopamine function in individuals with schizophrenia. There is no clear evidence of publication bias.

**
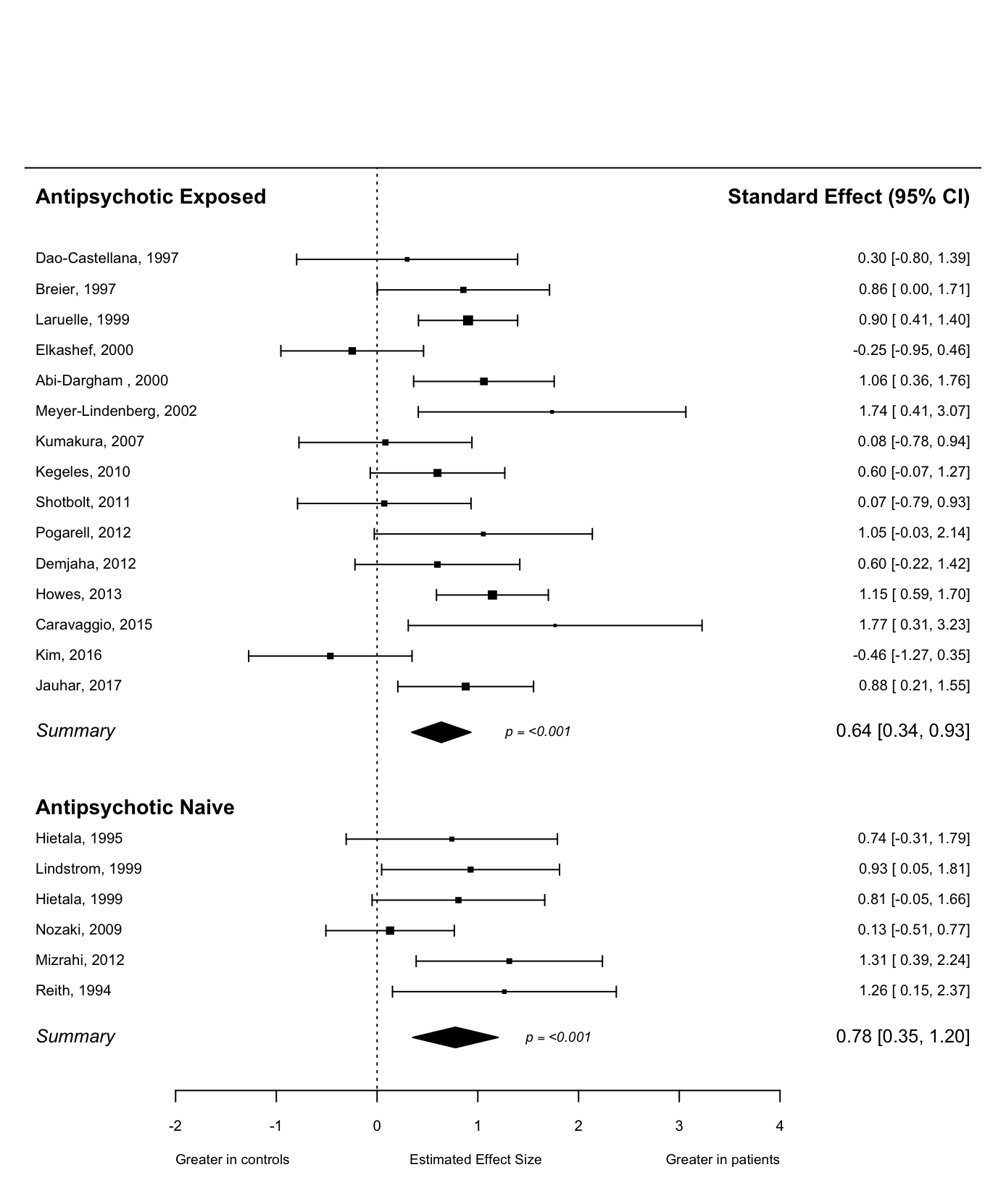
**

**eFigure 4.** Studies of presynaptic dopamine function in individuals with schizophrenia. Studies meta- analyzed separately depending on whether patients antipsychotic exposed or predominantly naïve (≥75% patients naïve). Significant patient-control differences seen both in individuals who have been exposed to antipsychotics (g=0.65, P<0.001) and antipsychotic naïve individuals (g=0.78, P<0.001).


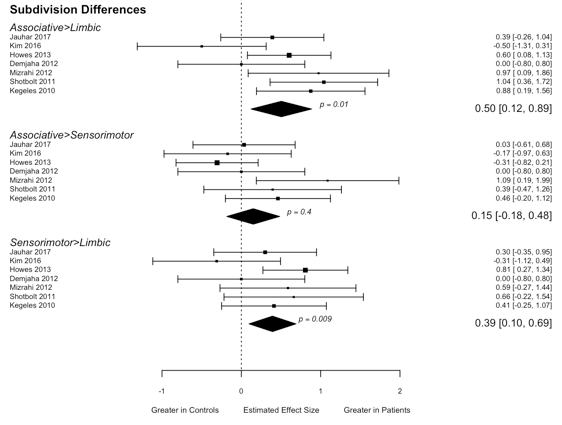


**eFigure5** Forest plot of subdivision differences in schizophrenia where a inter-subdivision correlation coefficient of 0.87 is used. Effect sizes for patient-control differences are increased (d=0.50 for associative>limbic comparison, and d=0.39 for sensorimotor>limbic comparison).

**eFigure6** Studies of presynaptic dopamine function in individuals with schizophrenia. Studies meta-analysed separately depending on whether a challenge/depletion paradigm or labelled L-DOPA used to index dopamine function. Significant patient-control differences were seen in both types of study (p<0.001 for each), although the summary effect size was significantly greater for the challenge/depletion studies compared to the DOPA studies (p=0.046).


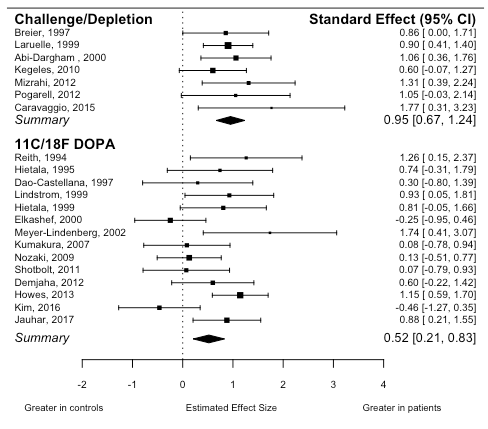


**References**

1. Howes O, McCutcheon R, Stone J. Glutamate and dopamine in schizophrenia: An update for the 21st century. *J Psychopharmacol*. 2015;**29**(**2**):97–115.

2. Howes O, Egerton A, Allan V. Mechanisms underlying psychosis and antipsychotic treatment response in schizophrenia: insights from PET and SPECT imaging. *Curr Pharm Des*. 2009;**15**(**22**):2550–2559.

3. Egerton A, Mehta MA, Montgomery AJ, et al. The dopaminergic basis of human behaviors: A review of molecular imaging studies. *Neurosci Biobehav Rev*. 2009;**33**(**7**):1109–1132.

4. Laruelle M, Abi-dargham A, Dyck CH Van, et al. Single photon emission computerized tomography imaging of schizophrenic subjects. *Proc Natl Acad Sci*. 1996;**93**:9235–9240.

5. Breier A, Su TP, Saunders R, et al. Schizophrenia is associated with elevated amphetamine-induced synaptic dopamine concentrations: evidence from a novel positron emission tomography method. *Proc Natl Acad Sci U S A*. 1997;**94**(**6**):2569–74.

6. Mizrahi R, Addington J, Rusjan PM, et al. Increased stress-induced dopamine release in psychosis. *Biol Psychiatry*. 2012;**71**(**6**):561–567.

7. Abi-Dargham A, van de Giessen E, Slifstein M, Kegeles LS, Laruelle M. Baseline and amphetamine-stimulated dopamine activity are related in drug-naïve schizophrenic subjects. *Biol Psychiatry*. 2009;**65**(**12**):1091–3.

8. Kegeles LS, Abi-Dargham A, Frankle WG, et al. Increased synaptic dopamine function in associative regions of the striatum in schizophrenia. *Arch Gen Psychiatry*. 2010;**67**(**3**):231–9.

9. Howes OD, Kambeitz J, Stahl D, et al. The Nature of Dopamine Dysfunction in Schizophrenia and What This Means for Treatment. *Arch Gen Psychiatry*. 2012;**69**(**8**):776–786.

10. Tziortzi AC, Searle GE, Tzimopoulou S, et al. Imaging dopamine receptors in humans with [11C]-(+)-PHNO: Dissection of D3 signal and anatomy. *Neuroimage*. 2011;**54**(**1**):264–277.

11. Ku HH. Notes on the use of propagation of error formulas. *J Res Natl Bur Stand Sect C Eng Instrum*. 1966;**70C**(**4**):263.

12. Borenstein M, L.V. H, Higgins JPT, Rothstein HR. *Introduction to Meta-Analysis - Complex Data Structures*. John Wiley & Sons; 2009.

13. Jauhar S, Nour MM, Veronese M, et al. A Test of the Transdiagnostic Dopamine Hypothesis of Psychosis Using Positron Emission Tomographic Imaging in Bipolar Affective Disorder and Schizophrenia. *JAMA Psychiatry*. 2017.
